# Rationale and design of a randomized trial of early intensive blood pressure lowering on cerebral perfusion parameters in thrombolysed acute ischemic stroke patients

**DOI:** 10.1097/MD.0000000000012721

**Published:** 2018-10-05

**Authors:** Vijay K. Sharma, Benjamin Y.Q. Tan, M. Ying Sim, Amit Kulkarni, Philip A. Seow, Chiew S. Hong, Zhengdao Du, Lily Y.H. Wong, Jintao Chen, Elaine Y.H. Chee, Bridget S.M. Ng, Yingliang Low, Nicholas J.H. Ngiam, Leonard L.L. Yeo, Hock L. Teoh, Prakash R. Paliwal, Rahul Rathakrishnan, Arvind K. Sinha, Bernard P.L. Chan, Kenneth Butcher, Craig S. Anderson

**Affiliations:** a Division of Neurology, Department of Medicine, National University Health System; b Yong Loo Lin School of Medicine, National University of Singapore; c Department of Diagnostic Imaging, National University Health System, Singapore, Singapore; d University of Alberta, Edmonton, Canada; e The George Institute for Global Health, Faculty of Medicine, University of New South Wales, NSW, Australia; f The George Institute China at Peking University Health Science Center, Beijing, PR China.

**Keywords:** acute ischemic stroke, blood pressure, cerebral blood flow, computed tomography perfusion, thrombolysis

## Abstract

**Background and Rationale:**

Uncertainty persists over the optimal management of blood pressure (BP) in the early phase of acute ischemic stroke (AIS). This study aims to determine the safety and effects of intensive BP lowering on cerebral blood flow (CBF) and functional in AIS patients treated with intravenous thrombolysis.

**Methods:**

In a randomized controlled trial, 54 thrombolysed AIS patients with a systolic BP of 160 to 180 mm Hg will be randomized to early intensive BP lowering (systolic target range 140–160 mm Hg) or guideline-based BP management (systolic range 160–180 mm Hg) during first 72-hours using primarily intravenous labetalol. We hypothesize that early intensive BP lowering will not reduce CBF by 20% and/or increase the volume of hypoperfused tissue by >20% on computed tomographic perfusion. Clinical outcome will be assessed using a dichotomized modified Rankin scale (scores 0–1 as excellent outcome vs scores 2–6 as dead or dependent) at 90 days. Other outcome would be symptomatic intracerebral hemorrhage. The trial is registered at ClinicalTrials.gov, NCT03443596.

**Conclusion:**

This randomized study will provide important information about the physiological effects of BP reduction on cerebral perfusion after intravenous thrombolysis in AIS.

## 1 Introduction and rationale

Elevated systolic blood pressure (BP), defined as >140 mm Hg, is commonly observed in patients with acute ischemic stroke (AIS).^[1]^ Potential mechanisms and associations include pre-existing hypertension,^[1]^ activation of neuroendocrine systems,^[2]^ stress of hospitalization,^[3]^ location of the infarct,^[4]^ stroke subtype,^[5]^ severity,^[6]^ and a homeostatic response to raised intracranial pressure.^[7]^ Whether treatment of this acute hypertensive response improves clinical outcome is unknown.^[7,8]^ Arguments in favor of treatment include the strong positive relationship between degree of elevation in BP and death or dependency,^[9]^ cerebral edema,^[10]^ early and late stroke recurrence,^[11,12]^ and extrapolation of potential benefits in acute intracerebral hemorrhage (ICH).^[13–15]^ Arguments against treatment center on the natural tendency for BP to normalize within a few days of presentation,^[1,5,6]^ and potential harms associated with altered cerebral perfusion related to dysfunction of autoregulation.^[7,16,17]^ Some studies suggest that adverse outcomes are associate with early antihypertensive therapy.^[18,19]^

The primary aim of the study is to determine the effect of early intensive BP lowering (systolic BP target 140–160 mm Hg) initiated within 6 hours of symptom onset and maintained for 72 hours in AIS patients treated with intravenous tissue plasminogen activator (IV-tPA) on cerebral blood flow (CBF), measured with computed tomographic (CT) perfusion imaging. We hypothesize that this treatment approach is safe and that such magnitude of BP lowering does not reduce CBF by 20% and/or increase the volume of hypoperfused tissue by >20% on CT perfusion or adversely affect functional outcomes at 90 days. Secondary aims are to obtain preliminary estimates of the clinical impact of early intensive BP lowering on functional outcomes and symptomatic intracerebral hemorrhage (sICH).

## 2 Methods

### 2.1 Patients

Patient inclusion/exclusion criteria are provided in Table 1. Adult patients with AIS aged 21 years and more (being the age of majority in Singapore) presenting to a single tertiary care hospital treated with IV-tPA as a standard-of-care are eligible for inclusion in this study if systolic BP is elevated (160–180 mm Hg) within 8 hours of symptom onset. Informed consent must be obtained from the patient or their legally authorized representative. Exclusion criteria include a documented allergy to a radiocontrast agent, significant renal impairment (defined with serum creatinine >176 μmol/L or estimated glomerular filtration rate <30 mL/minute), congestive cardiac failure, or allergy to labetalol, the principle IV antihypertensive agent used in the study.

**Table 1:**
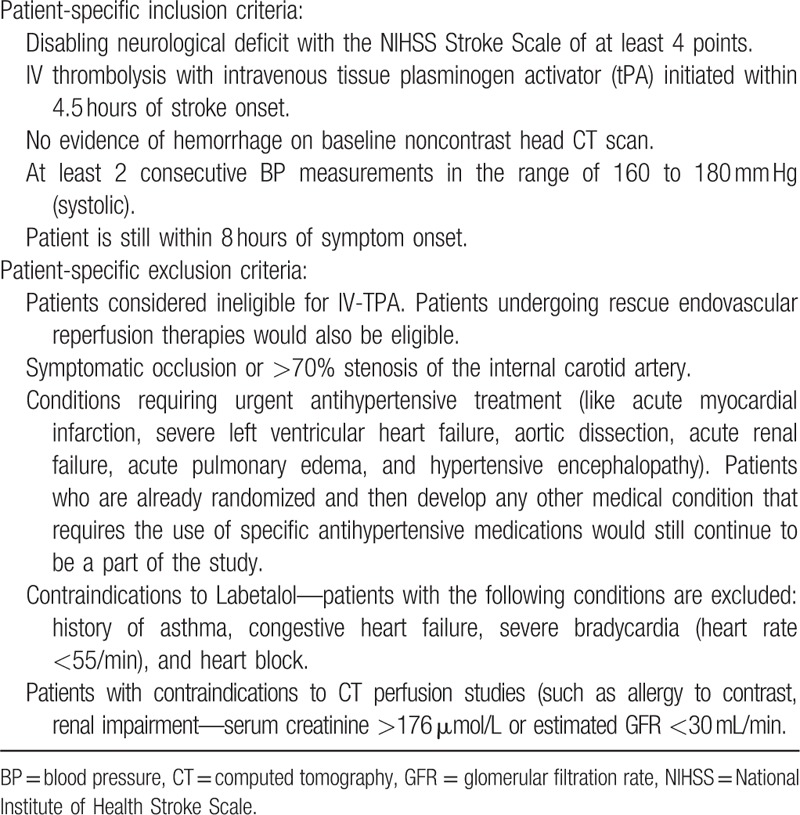
Inclusion/exclusion criteria for the trial.

### 2.2 Outcome measures

We reiterate that all patients included in this clinical trial would have received IV-tPA as standard of care before assessing their eligibility. All patients undergo a baseline clinical evaluation that includes an assessment of severity of neurological impairment according to the National Institutes of Health Stroke Scale (NIHSS), and estimate of their premorbid functional status [according to the modified Rankin scale (mRS); scores 0–2 required for inclusion]. All of them would have undergone a noncontrast brain CT-scan (or magnetic resonance imaging) with or without angiography as per the institutional practice. Information for demographics, risk factors (hypertension, diabetes mellitus, dyslipidemia, ischemic heart disease, smoking, and atrial fibrillation) and use of antihypertensive medications is also collected.

### 2.3 Randomization and blinding

All study subjects undergo CT perfusion to obtain the baseline cerebral hemodynamic parameters. Participating patients are randomly assigned to early intensive BP lowering (140–160 mm Hg) or the guideline-based management (160–180 mm Hg) in a 1:1 ratio using a central computer generated sequence with minimization according to age (<60 vs ≥60 years), severity (NIHSS score <10 vs >10), and baseline systolic B (<150 vs >150 mm Hg). The treating physician is unblinded to the treatment assignment as he/she is required to titrate BP control with IV labetalol. However, all 90-day outcome assessments are conducted by physicians who are kept blind to the treatment allocation.

### 2.4 Intervention

Patients allocated to early intensive BP lowering are to receive IV labetalol according to a predefined protocol (Table 2), whereas those assigned guideline-based BP management receive current standard of care. All study participants are closely monitored and a CT perfusion scan is repeated at 24 and 72 hours postrandomization. The treatment regime is an initial IV bolus (5–10 mg each, repeated at 10-minute intervals, maximum dose 80 mg) followed by continuous IV infusion to maintain the desired BP.

**Table 2:**
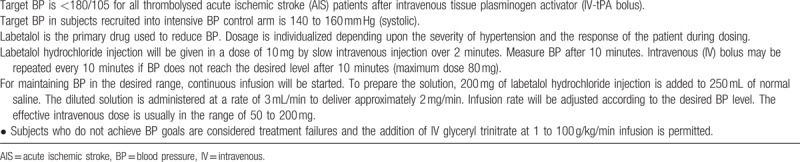
Protocol for blood pressure control in thrombolysed acute ischemic stroke patients.

### 2.5 Background care

**Figure 1:**
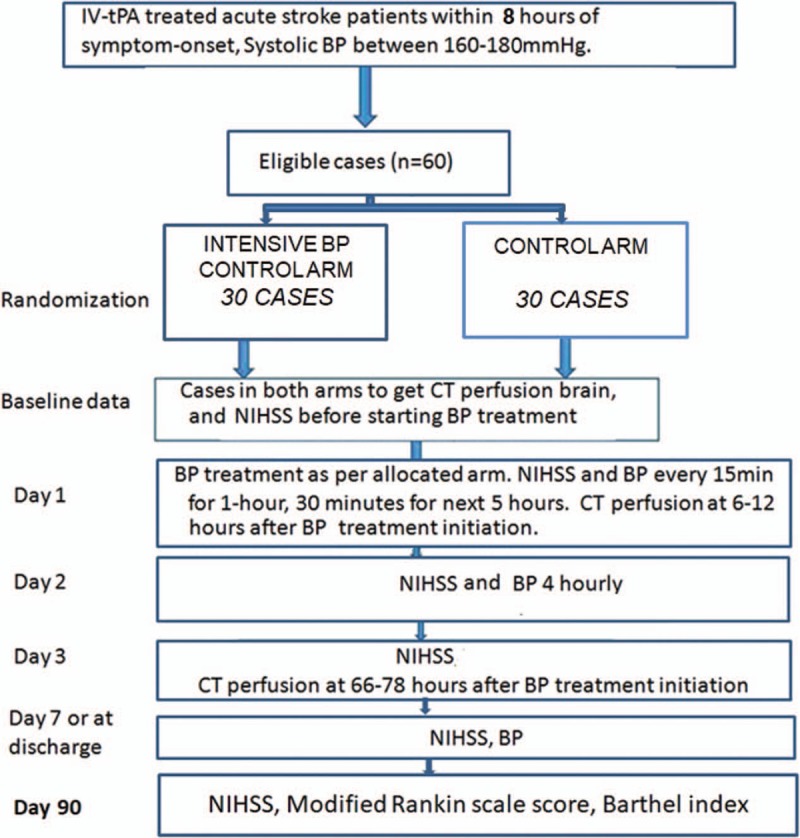
Study workflow. BP = blood pressure, CT = computed tomographic, IV-tPA = intravenous tissue plasminogen activator, NIHSS = National Institutes of Health Stroke Scale.

All patients will be managed in a high-dependency stroke unit with repeated neurological examination using the NIHSS, noninvasive BP, and heart rate monitoring (consistent recordings using automatic devices, every 15 minutes for 1 hour, then hourly for 23 hours, then 6 hourly until discharge). All BP measurements are obtained from the nonparetic arm. Rescue endovascular intervention (before inclusion into the study and randomization) is allowed according to local institutional practice. Figure 1 describes the workflow in the study.

### 2.6 Data collection and follow-up

All patients undergo repeat CT-perfusion scans at 24 and 72 hours postrandomization, to obtain the estimates of the effects of BP on various cerebral perfusion parameters. NIHSS scores are assessed daily until day 7 (or hospital discharge, if earlier) (Table 2). Patients are followed-up at day 90, where the NIHSS and mRS scores are reassessed. These assessments are conducted in-person by trained staff who are blind to the treatment allocation.

### 2.7 Outcomes

The primary outcome is the assessment of CT perfusion studies and evaluate whether CBF in the affected region reduces by >20% and/or the volume of hypoperfused tissue increases by >20% if BP is treated intensively during the first 72 hours in thrombolysed AIS patents. Secondary endpoint is dependency at day-90, defined by scores 2 to 6 on the mRS. Secondary outcome would be sICH, defined by the Safe Implementation of Thrombolysis in Stroke-Monitoring Study (SITS-MOST) criteria as large local or remote parenchymal ICH (type 2) combined with a neurological deterioration (≥4 points on the NIHSS) from baseline or death within 36 hours.^[20]^ Data are entered prospectively on to case report forms without any personal identifiers.

### 2.8 Data monitoring body

An independent data safety monitoring board is in place to monitor the safety of intensive BP lowering as well as repeated CT perfusion studies. The board reviews clinical and imaging data in batches of 10 patients each. Unexplained clinical deterioration in intensive BP control arm is reviewed in a multidisciplinary meeting (neurologists, neurosurgeons, neuroradiologist, and interventional neuroradiologists). Treating stroke neurologist is allowed to discontinue intensive BP lowering treatment (if the subject is allocated to this treatment arm), if this is considered to be the sole/leading cause of neurological deterioration. Radiation safety officer will be using the real patient data as well as a phantom to monitor radiation exposure to brain, eyes, and thyroid gland. For reassurance, we used a phantom and evaluated the radiation dose exposure for one non-contrast CT (NCCT) + CT angiogram + CT perfusion (CTP), which was quite low as 6.13 mSv.

### 2.9 CT perfusion acquisition

With dynamic, quantitative CT perfusion, an additional contrast bolus of 35 to 45 mL is administered via an injector (at a rate of 7 mL/s), with a saline “chaser” of 20 to 40 mL at the same injection rate. The contrast used (omnipaque) has high concentration, typically 350 to 370 g/dL for optimal signal-to-noise ratio for perfusion maps calculation. Imaging begins a few seconds after injection, and selective axial sections to image selected areas of the brain using “toggle mode” expands the anatomic coverage by toggling the table between 2 locations during dynamic scanning of 4 cm cuts of the brain. A Philips 64 slice scanner with standard resolution is used with collimation 32 × 1.25, thickness 5 mm, 3.8 seconds cycle time, 80 kV at 150 mA, matrix 512, and window-center/width of 40/80. Scans take 55 to 60 seconds to complete 15 jog cycles.

### 2.10 CT perfusion image analysis

Perfusion images will be analyzed centrally by investigators blinded to BP treatment and clinical data. Raw CTP source images will be transferred to a computer workstation and postprocessed using the MIStar software package, version 3.2 (Apollo Medical Imaging Technology, Melbourne, Australia). An arterial input function will be selected automatically in the contralateral middle cerebral artery. An arrival time insensitive singular value decomposition, deconvolution algorithm will be used to calculate voxel-wise maps of CBF, cerebral blood volume (CBV), mean transit time (MTT), and delay time to peak of the residual function (DT).^[16]^ The total perfusion deficit volume will defined using a threshold DT >6 seconds (DT). The pretreatment (baseline) ischemic core will be defined as tissue with a CBF of <30% inside the perfusion deficit. The penumbra will be defined as tissue within the DT deficit which was not the ischemic core [i.e., relative CBF (rCBF) >30%]. Hypoperfused tissue, penumbral, and core volumes will be measured at all 3 time points.^[17]^

Final infarct regions of interest will be drawn on the 24 hour NCCT and coregistered to CBF, CBV, MTT, and DT maps obtained at all 3 time points. The mean absolute perfusion value within each region will then be measured. Mean perfusion values will also be measured in homologous regions of the contralateral hemisphere. rCBF, relative CBV, and relative MTT will be calculated as the ratio of the absolute mean value in the infarct core and homologous contralateral region. Relative DT will be defined as the difference between the absolute mean in infarct region and that in the contralateral homologous region.

### 2.11 Sample size calculations

Allowing for within-subject and between-subjects variation, a sample size of 27 patients per group was estimated to allow detection of a difference of 20% [15% standard deviation (SD)] in relative change in CBF and/or the volume of hypoperfused tissue between the early intensive BP lowering and control groups, with 90% power and significance set at 5% (α = 0.050, 2-tailed). The sample size was increased to 30 patients per group to allow for treatment dropouts, crossovers, and potential loss to follow-up.

### 2.12 Statistical analysis

Data will be analyzed blind to treatment group according to the intention-to-treat principle by an independent biostatistician. Continuous variables will be presented as mean (SD) and nominal variables tabulated as absolute and/or relative frequencies. Comparisons between binary outcomes will be performed using Fisher exact test. Continuous variables will be compared using the unpaired *t* test and Mann-Whitney *U* test as indicated. Comparisons of BP and NIHSS scores between the 2 groups at different time-points will be analyzed using repeated-measures analysis of variance for the overall effect. Finally, the relative changes in BP, CBF, and NIHSS scores at 1, 24, and 72 hours after the initiation of treatment, and 90-day outcomes, will be compared using analysis of covariance. Baseline BP and use of antihypertensive medications before admission will be used as covariates in analyses of BP differences. Baseline NIHSS will be used as a covariate in analyses of stroke severity. Statistical significance is set at the 5% level. There would be no interim analysis (Table 3).

**Table 3:**
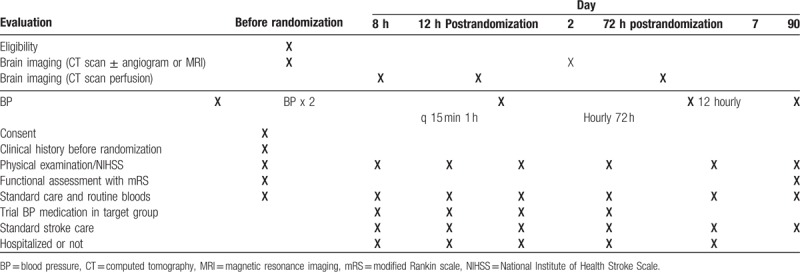
Schedule of evaluations during the study.

### 2.13 Funding and ethics approval

This study is funded by the National Medical Research Council, Singapore as a research grant as part of a Clinician Scientist Award to Dr Vijay K. Sharma. The study has been approved by the institutional ethics committee. The trial is registered at ClinicalTrials.gov, NCT03443596.

## 3 Discussion

As a U-shaped relationship of systolic BP and outcomes after IV-tPA is suggested in various studies,^[8,10,12]^ both high and low systolic BP are considered harmful. Interestingly, the nadir for optimal outcome appears to be a systolic BP of 140 to 160 mm Hg. Thus, we hypothesize this systolic BP as being the best target for functional outcome and reduced risk of sICH.

Labetalol was chosen for BP lowering because of ready availability, minimal adverse effects on heart rate, and cardiac output due to being a mixed α and β antagonist effects, rapid onset of action (5 minutes) and reasonable duration of action (3–6 hours). Studies indicate stable effects of labetalol on CBF, cerebral oxygen consumption, and cerebral circulation.^[21,22]^ There is good experience of the use of labetalol in patients with AIS^[23]^ and it has a predictable and uniform dose-response relationship.^[23]^

With serial BP measurements (including comparison of different intensities of BP lowering), NIHSS and repeated CT perfusions, this study will provide mechanistic data to elucidate the relationship between BP, cerebral perfusion, and functional outcomes in thrombolysed AIS cases. Our findings may assist the ongoing studies (such as ENCHANTED BP intensity arm results due in early 2019) and improve the design of future clinical trials of BP management in AIS (in thrombolysed and endovascularly treated) patients.

Finally, we wish to clarify that study is not aimed at evaluating the relationship between early intensive BP reduction and arterial recanalization or persisting occlusion. Study participants are recruited after the arterial recanalization treatment strategies (IV thrombolysis and/or EVT) have already been implemented. While the first CT perfusion serves as the baseline for a particular patient, subsequent perfusion scans provide information about the longitudinal changes in cerebral hemodynamic parameters in response to the changes in BP levels.

## Author contributions

Vijay K. Sharma, Benjamin Y.Q. Tan, and Sim M. Ying were responsible for concept of the study, writing the draft, and editing of the manuscript. Amit Kulkarni, Philip A. Seowa, Chiew S. Hong, Zhengdao Du, Lily Y.H. Wong, Jintao Chen, Elaine Y.H. Chee, and Bridget S.M. Ng were responsible for data gathering. Low Yingliang and Nicholas J.H. Ngiam were responsible for data analysis. Hock L. Teoh, Prakash R. Paliwal, Rahul Rathakrishnan, Arvind K. Sinha, and Bernard P.L. Chan were responsible for editing the manuscript. Kenneth Butcher and Craig S. Anderson provided critical appraisal and editing of the manuscript.

**Conceptualization:** Vijay K. Sharma, Benjamin Y.Q. Tan, Hock L. Teoh.

**Data curation:** Vijay K. Sharma, Amit Kulkarni, Chiew S. Hong, Zhengdao Du, Elaine Y.H. Chee, Bridget S.M. Ng, Low Yingliang, Nicholas J.H. Ngiam, Rahul Rathakrishnan, Arvind K. Sinha.

**Formal analysis:** Amit Kulkarni, Bridget S.M. Ng, Low Yingliang, Nicholas J.H. Ngiam, Arvind K. Sinha.

**Funding acquisition:** Vijay K. Sharma.

**Investigation:** Vijay K. Sharma, Low Yingliang, Prakash R. Paliwal, Rahul Rathakrishnan, Bernard P.L. Chan.

**Methodology:** Vijay K. Sharma.

**Project administration:** Vijay K. Sharma, Philip A. Seow, Zhengdao Du, Lily Y.H. Wong, Jintao Chen, Prakash R. Paliwal.

**Resources:** Philip A. Seow, Jintao Chen.

**Software:** Kenneth Butcher.

**Supervision:** Leonard L.L. Yeo, Hock L. Teoh, Prakash R. Paliwal, Bernard P.L. Chan, Craig S. Anderson.

**Writing – original draft:** Benjamin Y.Q. Tan, Sim M. Ying.

**Writing – review and editing:** Leonard L.L. Yeo, Kenneth Butcher, Craig S. Anderson.
